# Efficacy and safety of Suxiao Jiuxin Pill in the treatment of stable angina (Qi stagnation and blood stasis syndrome): study protocol of a randomized, double-blind, placebo-controlled, multi-center clinical trial

**DOI:** 10.1186/s13063-021-05448-6

**Published:** 2021-07-19

**Authors:** Xiaofen Ruan, Yiping Li, Yuanlong Sun, Meijun Jia, Xiaowen Xu, Li Huo, Wei Song, Yili Yao, Xiaolong Wang

**Affiliations:** 1 Cardiovascular Department, Shuguang Hospital of Shanghai University of Traditional Chinese Medicine, No. 528 Zhangheng Road, Pudong New Area, Shanghai, 201203 China; 2 Cardiovascular Research Institute of Traditional Chinese Medicine, Shuguang Hospital of Shanghai University of Traditional Chinese Medicine, Shanghai, 201203 China; 3 Institute of Liver Disease, Shuguang Hospital of Shanghai University of Traditional Chinese Medicine, Shanghai, 201203 China

**Keywords:** Stable angina, Qi stagnation and blood stasis syndrome, Suxiao Jiuxin Pill, Traditional Chinese medicine

## Abstract

**Background:**

Coronary heart disease (CHD) has become one of the biggest health problems in the world. Stable angina is a common clinical type of CHD with poor prognosis and high mortality. Although there are various interventions for stable angina, none of them can significantly reduce mortality. Both basic and clinical research have shown that Suxiao Jiuxin Pill (SJP) can relieve the symptoms of angina pectoris and improve the clinical efficacy, but there is a lack of high-quality clinical research to provide research-based evidence. We design a randomized, double-blind, placebo-controlled trial to evaluate the efficacy of SJP for stable angina.

**Methods/design:**

This is a prospective, randomized, double-blind, placebo-controlled, and multicenter trial. The trial will enroll 324 participants with chronic stable angina (Qi Stagnation and Blood Stasis syndrome). All participants will have received the conventional therapy of chronic stable angina. Participants will be randomized into two groups, conventional therapy plus SJP group and conventional therapy plus placebo group. Eligible participants will receive either SJP or placebo (five pills administered orally, three times daily) in addition to conventional treatment for 24 weeks. The primary outcomes are the symptom improvement rate of angina from baseline to 4 weeks after inclusion and major adverse cardiovascular events (MACE). The secondary outcomes are angina classification (CCS), improvement of traditional Chinese medicine (TCM) syndromes, Seattle Angina Scale score, the dosage of emergency drugs and the stopping rate, and electrocardiogram (EKG) efficacy. Adverse events will be monitored throughout the trial.

**Discussion:**

Integrated traditional Chinese and Western Medicine is commonly used for angina in China. This study will evaluate the clinical effectiveness and safety of SJP for angina. The results of the trial will provide high-level clinical research-based evidence for the application of SJP instable angina.

**Trial registration:**

This study protocol was registered on 14 March 2019. The registration number is ChiCTR1900021876 on the Chinese Clinical Trial Registry.

**Supplementary Information:**

The online version contains supplementary material available at 10.1186/s13063-021-05448-6.

## Administrative information

Note: the numbers in curly brackets in this protocol refer to Additional file 1: SPIRIT checklist item numbers. The order of the items has been modified to group similar items (see http://www.equator-network.org/reporting-guidelines/spirit-2013-statement-defining-standard-protocol-items-for-clinical-trials/).

**Table:** 

**Title {1}**	**Efficacy and safety of Suxiao Jiuxin Pill in the treatment of stable angina (Qi stagnation and blood stasis syndrome): Study protocol of a randomized, double-blind, placebo-controlled, multi-center clinical trial**
**Trial registration {2a and 2b}.**	ChiCTR1900021876, ChiCTR
**Protocol version {3}**	3-14-2019, version 1.0
**Funding {4}**	(1) National Outstanding Youth Science Fund Project of National Natural Science Foundation of China. Award Number: 81403352 | Recipient: Xiaofen Ruan (2) National Outstanding Youth Science Fund Project of National Natural Science Foundation of China. Award Number: 81803887 | Recipient: Yiping Li (3) Shanghai Promoting TCM 3-Year Action Program (ZY(2018-2020)-RCPY-2004) | Recipient: Xiaofen Ruan
**Author details {5a}**	(1) Xiaofen Ruan, Cardiovascular Department, Shuguang Hospital of Shanghai University of Traditional Chinese Medicine, Shanghai, China; ruanxiaofeng@shutcm.edu.cn (2) Yiping Li, Cardiovascular Department, Shuguang Hospital of Shanghai University of Traditional Chinese Medicine, Shanghai, China; liyiping@shutcm.edu.cn (3) Yuanlong Sun, Cardiovascular Department, Shuguang Hospital of Shanghai University of Traditional Chinese Medicine, Shanghai, China; 823951231@qq.com (4) Meijun Jia, Cardiovascular Department, Shuguang Hospital of Shanghai University of Traditional Chinese Medicine, Shanghai, China; 1852jmj@shutcm.edu.cn (5) Xiaowen Xu, Cardiovascular Department, Shuguang Hospital of Shanghai University of Traditional Chinese Medicine, Shanghai, China; 2428420670@qq.com (6) Li Huo, Cardiovascular Department, Shuguang Hospital of Shanghai University of Traditional Chinese Medicine, Shanghai, China; hl970204@163.com (7) Wei Song, Cardiovascular Department, Shuguang Hospital of Shanghai University of Traditional Chinese Medicine, Shanghai, China; swei222@shutcm.edu.cn (8) Yili Yao, Cardiovascular Department, Shuguang Hospital of Shanghai University of Traditional Chinese Medicine, Shanghai, China; yao_stephanie@shutcm.edu.cn (9) Xiaolong Wang, Cardiovascular Department, Shuguang Hospital of Shanghai University of Traditional Chinese Medicine, Shanghai, China; wxlqy0214@163.com
**Name and contact information for the trial sponsor {5b}**	Sponsor: Shuguang Hospital of Shanghai University of Traditional Chinese Medicine, Zhangheng Road 528, Pudong New Area, Shanghai, China Coordinating Investigator (contact): Prof. Dr. Xiaolong Wang Cardiovascular Department Shuguang Hospital of Shanghai University of Traditional Chinese Medicine Zhangheng Road 528 Pudong New Area, Shanghai, China Tel: 086-13501991450 Email: wxlqy0214@163.com
**Role of sponsor {5c}**	The sponsor and the funding body have no role in the design of the study, in collection, analysis and interpretation of the data and in the writing of the manuscript

## Background

Coronary heart disease (CHD) stable angina pectoris is also known as stable labor angina pectoris, which refers to the clinical syndrome caused by myocardial ischemia and hypoxia due to coronary atherosclerotic stenosis or thrombosis and spasm. Specifically, it is defined such that its clinical manifestations are relatively stable within 1 to 3 months, i.e., the number of daily and weekly pain episodes is approximately the same, the degree of labor and emotional agitation to induce pain are the same, the nature and location of pain do not change during each episode, and the pain duration is similar. It has characteristics, such as high mortality and many complications. The prevalence and mortality of cardiovascular disease (CVD) in China are continuously rising [1]. The prevalence of angina pectoris in China is 3.6% [2]. In the USA, about 10 million people suffer from angina pectoris and more than 500,000 new cases are diagnosed each year [3]. The annual mortality rate of stable angina is as high as 3.2% in Europe [4]. The aims of treatment for angina include controlling the clinical symptoms and preventing cardiovascular events. Western medicine treatments include antiplatelet therapy (APT), angiotensin-converting enzyme inhibitor (ACEI) or angiotensin II receptor antagonist (ARB), beta-blockers, calcium channel blockers, nitrate, and statins [5]. However, many participants continue to suffer from angina. Angina worsening and repeated hospitalization has caused severe economic burden on families and society. Supplementary replacement therapy is necessary for the treatment of angina. Integrated traditional Chinese and Western Medicine is commonly used for angina in China.

Suxiao Jiuxin Pill (SJP, produced by Tianjin Zhongxin Pharmaceutical Group Co, Ltd.) is a patented drug made of active ingredients from Chuanxiong and Borneol, and it has the effect of regulating Qi and promoting blood circulation. SJP can increase coronary blood flow, improve myocardial cell hypoxia, and relieve angina pectoris similar to nitroglycerin [6, 7]. SJP has been commonly used in the treatment of angina pectoris with integrated Chinese and Western medicine, and these participants have also been diagnosed with Qi stagnation and blood stasis syndrome [8–10]. Experimental research has confirmed that SJP could dilate the blood vessels by regulating [Ca2+], increase myocardial blood supply, and promote cardiomyocyte proliferation in vitro and in vivo [11, 12]. Several clinical researches on SJP have confirmed its efficacy in mitigating the symptoms of angina without any serious side effects [13, 14]. However, the evidence remains weak due to the poor methodological quality of included studies [7]. The purpose of this study is to clarify the efficacy and safety of SJP in the treatment of angina pectoris through a high-quality clinical experimental design. The experimental results will provide high-level research-based evidence for the clinical application of SJP.

## Methods/design

### Study objectives

To evaluate the effectiveness and safety of SJP in patients with chronic stable angina pectoris (Qi stagnation and blood stasis syndrome) based on standardized western treatment.

### Study design

The study is designed as a randomized, double-blind, multicenter, placebo-controlled trial. To avoid regional differences, the study will be conducted in 11 hospitals from different regions of China. Data management and statistical analysis will be performed by the statistical department in China Evidence-based Medicine Center, West China Hospital, Sichuan University. This study will be conducted by following the Declaration of Helsinki and the guidelines of Good Clinical Practice (GCP). The study has also been approved by the ethics committee in Shuguang Hospital (Ethics approval number: 2019-649-04-01). Trial registration: ChiCTR, ChiCTR1800014258. Registered on 13 March 2019 at chictr.org.cn (http://www.chictr.org.cn/showproj.aspx?proj=34955). Before randomization, all participants in this study must sign an informed consent form.

**Fig. 1:**
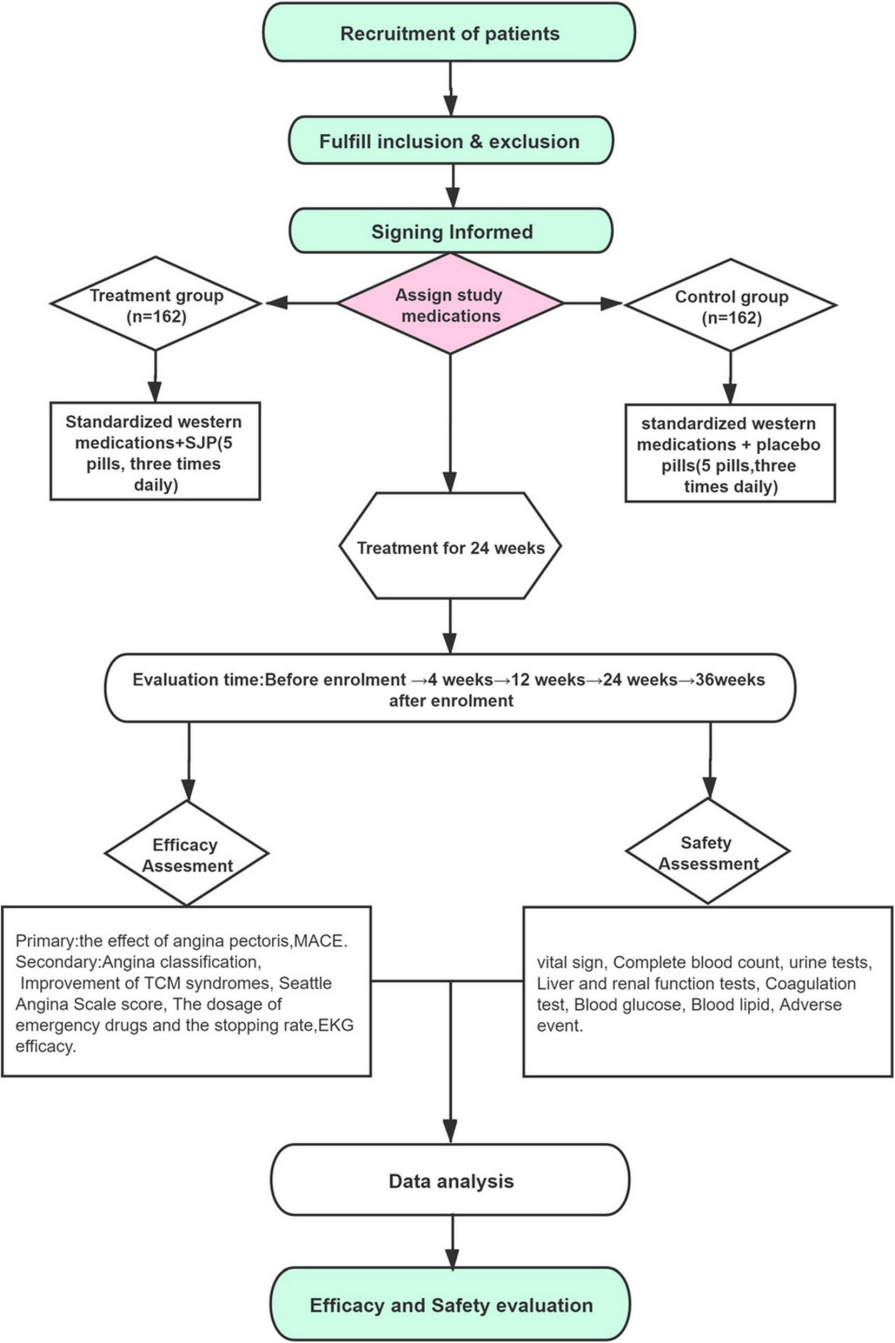
Flow diagram

The participants who fulfill both the inclusion and exclusion criteria will be recruited into the study. Participants sign consent form. Demographic information (including date of birth, gender, ethnicity, height, and weight), medical and treatment history (including course of disease, smoking, drinking history), angina pectoris score, Seattle angina pectoris scale score, physical and chemical examinations (EKG, blood lipids, C-reactive protein, tumor necrosis factor alpha, and interleukin 6) and scores from the four traditional Chinese medicine (TCM) diagnostic methods are collected. The study design is shown in Fig. 1.

### Eligibility criteria

Eligible patients will be those who fulfill all of the following inclusion criteria and who do not have any of the listed exclusion criteria.

#### Diagnostic criteria

1. Basis of diagnosis of CHD (diagnosis of any of the following)/The diagnostic criteria for CHD will be ① history of myocardial infarction (MI); ② coronary angiography or computed tomography coronary angiography confirmation of stenosis greater than 50%; and ③ noninvasive imaging stress test diagnostic of coronary artery disease (CAD) (e.g., positive treadmill exercise test (male only), nuclear perfusion scan). Patients will be considered to be diagnosed with CHD if they meet at least one of these criteria [4, 5, 15].
2. The angina pectoris severity classification diagnostic criteria were formulated by following the “ACC/AHA /ACP-ASIM Chronic Stable Angina Pectoris Management Guidelines”. Canadian Cardiovascular Society (CCS) Angina Pectoris Severity Classification [16].

#### The standardization of TCM

According to the Expert Consensus for Diagnosis and Treatment of Coronary Heart Disease with Stable Angina Pectoris (2018) [17], doctors choose medications based on standardized western medications. The technical guidelines for clinical research of TCM drugs and natural drugs used for angina and coronary artery disease (2011) describe the main symptom as chest pain (chest tightness) [18]. The secondary symptoms are sighing and bloating. Tongue characteristic is a purplish tongue and pulse manifestation is a string or astringent pulse. A patient must have the main symptom and one of the secondary symptoms, in accordance with the tongue and pulse conditions, to be diagnosed with Qi stagnation and blood stasis syndrome [19].

#### Inclusion criteria

1. Age 18 to 80 years;
2. Diagnosed with CHD;
3. CCS classification of angina grades I–III;
4. Patients with angina pectoris twice or more per week;
5. TCM diagnosis of Qi stagnation and blood stasis syndrome; and
6. Submitted informed consent.

#### Exclusion criteria

1. Myocardial infarction in the past 3 months and moderate to severe heart failure, severe cardiopulmonary insufficiency;
2. Percutaneous coronary intervention or coronary artery bypass grafting or implantation of a pacemaker in the past 3 months;
3. Uncontrolled hypertension with systolic blood pressure (SBP) ≥ 180 mmHg or diastolic blood pressure (DBP) ≥ 100 mmHg, or severe arrhythmia;
4. Chest pain caused by other diseases, such as psychosis, severe neurosis, hyperthyroidism, biliary heart syndrome, gastroesophageal reflux, and aortic dissection;
5. Alanine transaminase and/or aspartate transaminase values two times higher than the upper reference limit value. Renal insufficiency (defined as serum creatinine value one and a half times higher than the upper reference limit value). Other severe primary diseases, such as hematopoietic system disease and malignant tumor;
6. Allergy to the ingredients of the study drug; and
7. Participation in other clinical trials in the past 1 month.

### Randomization

Eligible patients will be randomly assigned to the treatment group or the control group in a 1:1 ratio, using SAS 9.4 statistical software and a block randomization schedule. The size of the blocks is four. Randomization of the trial participants will be completed in an independent data center. Each participating hospital will have an independent drug administrator responsible for random coding and drug management. Each center set up an independent drug administrator, who was responsible for random coding and drug management.

### Blinding

Blind editing and blind bottom preservation: All patients and researchers will be blinded to the treatment assignments until the study is completed. Personnel unrelated to this clinical trial will complete the preparation of drug blinding and emergency letters. Duplicate blinding codes will be kept in the clinical research institution of drugs at Shuguang Hospital. The blinding codes will not be broken until all clinical data are entered into a database and locked, except in an emergency situation.

In this study, the data security monitoring committee decided whether the subjects who had security incidents needed to conduct emergency unblinding or discontinue the study. During the study period, once a safety event occurred to the participants, the preliminary assessment was conducted by the researchers, and the data security monitoring committee held a safety event assessment summary meeting every six months. The data management was performed by the third party certified by relevant departments, and the data acquisition and management were implemented by the validated EDC system.

### Interventions

Eligible patients will be randomized to either the treatment group or the control group. Based on the guidelines for diagnosis and treatment of chronic stability angina in China (2018) [5], doctors will give routine treatment. Both the experimental group and control group can take antiplatelet drugs, angiotensin-converting enzyme inhibitor (ACEI) or angiotensin II receptor antagonist (ARB), statins, calcium ions antagonists, beta-blockers, and long-acting nitrates; the above drugs can continue to be used without changing the original dose; nitroglycerin 0.5 mg can be taken sublingually when angina pectoris is intolerable; and detailed records of the frequency and the amount of medication. Other oral or intravenous traditional Chinese medicines that might potentially interfere with the therapeutic effects of SJP will not be allowed.

During the treatment period, eligible patients will be given SJP or placebo pills and they will have to take 5 pills of the study medication three times daily. SJP is composed of tetramethylpyrazine and borneol approval number Z12020025 of the China State Food and Drug Administration [SFDA]). The SJP and placebo pills are provided by Tianjin Zhongxin Pharmaceutical Group Co., Ltd. (Tianjin, China). The placebo pills are similar to the SJP, with a comparable appearance. Each pill weighs 40 mg. The primary ingredient of the placebo pills is starch. By adding flavoring agents and food colorants, the placebo pills achieve a texture, smell, color, and taste comparable to the contents of the SJP.

### Outcomes

The primary outcome is the effect in angina pectoris, a composite endpoint of cardiovascular events (cardiac death, nonfatal myocardial infarction, revascularization with PCI or coronary artery bypass graft, heart failure, and hospitalization for coronary heart disease). The secondary outcomes are angina classification, improvement of TCM syndromes, Seattle Angina Scale score, the dosage of emergency drugs and the stopping rate, EKG efficacy, and scores of the four TCM diagnostic methods.

### Follow-up and points of data capture

The points of data capture in the trial are as follows: Intervention period (24 weeks): Baseline, medication for 4 weeks ± 2 days, medication for 12 weeks ± 3 days, medication for 24 weeks ± 5 days for hospital visits, and telephone follow-up for 36 weeks ± 7 days after enrolment.

**Fig. 2:**
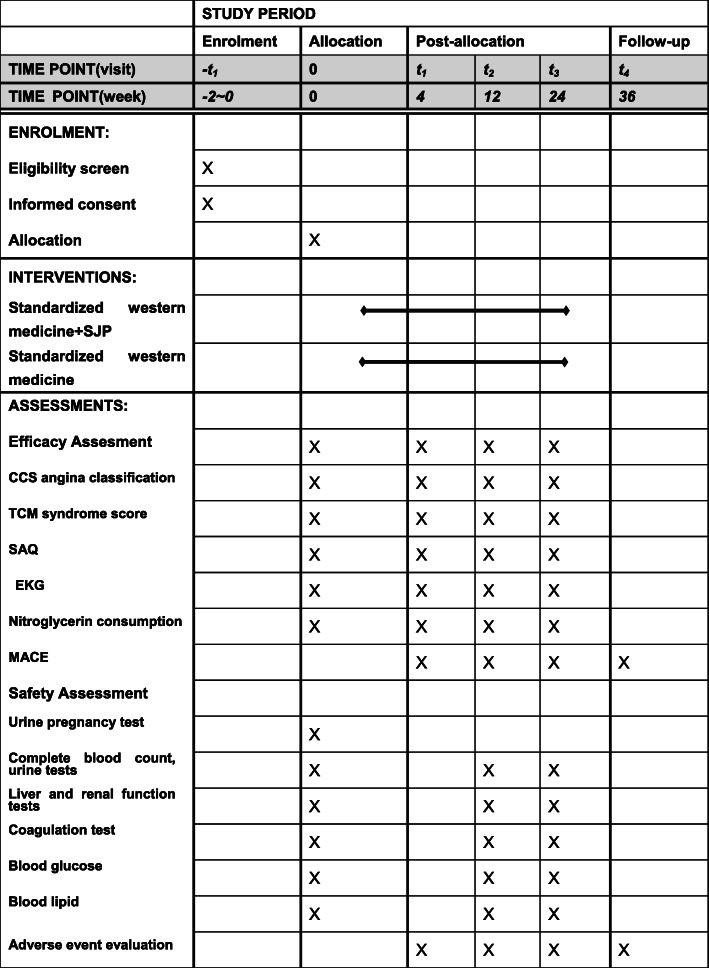
Study schedule (Standard Protocol Items: Recommendations for Interventional Trials, SPIRIT). Abbreviations: SJP, Suxiao Jiuxin Pill; CCS, Canadian Cardiovascular Society; TCM, Traditional Chinese medicine; SAQ, Seattle angina questionnaire; EKG, electrocardiogram; MACE, major adverse cardiac events

The corresponding items will be measured according to the time point of data collection. The detailed information is shown in Fig. 2.

### Sample size

The sample size will be calculated based on a 10% increase in effective treatment after treatment. It was hypothesized that the effective treatment rate for patients receiving standard western medicine treatment is 83% and that for patients receiving both traditional Chinese medicine and western medicine treatment is 93%. The participants will be arranged into the treatment or control group in a ratio of 1:1. According to the one-tailed difference test formula, there should be 162 participants in each group (significance level α is 0.05 and power (1-β) is 0.80 in the one-sided test) and the incomplete rate should be less than 20%. A total of 324 participants will be included.

### Statistical approach

Efficacy will be determined by the full analysis set (FAS) and per protocol set (PPS), while the safety assessment will be based on the safety set (SS). The statistical evaluation of FAS will follow the principle of intention to treat (ITT). For the missing values of the main efficacy evaluation indicators, the method of the last observation carried forward (LOCF) will be used. Continuous variables were shown as mean ± standard deviation (SD) and were compared using the *t* test or the nonpara-metric Mann-Whitney *U* test, as appropriate. The count variables were shown as the number of cases and percentage, and the chi-square test was used for comparative analysis. The symptom improvement rate of angina is to calculate the total score of angina pectoris according to the degree of pain, the number of attacks, and the duration. The total score is effective when it is lower than or equal to 30% of the baseline, and invalid when it is less than 30%. The symptom improvement rate of angina was analyzed by chi-square test. The participants who had any cardiovascular event during 24 weeks of treatment will be identified as the occurrence of cardiovascular events. The RR and 95% confidence interval of the two groups were calculated by logistic regression model. Statistical analysis will be performed using SAS 9.4 software.

### Data management and quality control

Any amendments or changes of protocol will be reapproved by the formal process of the ethics committee of Shuguang Hospital. Data management and statistical analysis will be performed by the statistical department in China Evidence-based Medicine Center, West China Hospital, Sichuan University. Independent clinical research associate (CRA) will regularly audit and monitor the study at each hospital. Well-trained researchers will collect and record the study data in the case report form (CRF). To ensure confidence in the data, study-related files including consent forms, the CRF, questionnaires, medical records, and other records will be stored in a locked space or on a password-protected computer in each hospital for 3 years after study completion. Data monitoring will be conducted by the data and safety monitoring committee. Auditing can be conducted to this trial by the quality assurance team at the supporting institution, independent from the investigators.

### Safety assessment

A safety analysis set will be used to summarize and analyze all safety endpoints. Safety assessment will be based on vital signs, laboratory examinations, and adverse events. Vital signs will include the respiratory rate, blood pressure, and heart rate. Laboratory measurements will include a routine blood and urine test, hepatorenal assessment, and electrocardiogram (detailed monitoring schedule is shown in Fig. 2). Adverse events, especially severe adverse events, will be reported to the research group committee within 24 h. The study design refers to Fig. 1. Based on the improvement of symptoms at 24 weeks, post-trial participants will be recommended to have a regular follow-up at the outpatient department.

## Discussion

Myocardial ischemia and hypoxia are critical pathophysiological changes in CHD. Although the treatment of angina pectoris is performed in accordance with the guidelines, many participants do not receive conventional treatments due to side effects, contraindications, and drug-drug interactions. Some participants receive conventional treatment; however, angina symptoms persist. CHD is called “chest pain,” “heartache,” and “true heartache” in the TCM concept [18]. The etiology and pathogenesis of TCM of CAD are deficient in origin and excess in superficiality. One of the main syndrome types is Qi stagnation and blood stasis syndrome. TCM has been used to treat diseases for thousands of years. Many classic formulas have been used in clinical practice. Recently, people are increasingly concerned about the benefits of TCM and potential drug interactions with Western medications, especially for participants with stable angina [15]. The combination of SJP and Western medicine is one conventional approach in the treatment of angina pectoris. Many experimental studies and clinical trials have proved the efficacy in the improvement of cardiocerebral vascular conditions [20–22]. The pathophysiology of SJP in the treatment of myocardial ischemia has not been fully elucidated. The main mechanisms include oxidative stress, calcium overload, the opening of mitochondrial permeability transition pore, and inflammation stimulation. The combination of Ligustrazine and Borneol (the main ingredients of SJP) showed a notable synergistic calcium antagonistic activity, exerting a better vasodilatation function than using any component alone [11]. Thus, it plays a protective role in myocardial ischemia.

Several clinical studies on SJP have confirmed its efficacy in mitigating the symptoms of angina without any serious side effects. A systematic review including 41 trials involving 6276 participants was included in the analysis. A meta-analysis of the clinical efficacy of the results. The analysis showed that SJP can effectively reduce the lipid profile and improve the hemorheology index in patients with CHD. This is due to the effects of its ingredients (borneol and L. chuanxiong), which may improve coronary artery circulation and ECG results. Based on the Jadad score, most of the clinical studies included in the meta-analysis were of poor quality. Only a few studies reported a detailed research methodology [13]. Therefore, it is necessary to scientifically design high-quality, large-scale, clinically proven therapeutic effects of fast-acting Jiuxin pills on angina. Evaluation of the therapeutic effect on cardiovascular disease includes improvement of clinical symptoms and long-term prognosis of the disease. Therefore, in addition to the efficacy in angina pectoris, the main therapeutic index of this experiment also observed the composite endpoint of cardiovascular events (MACE). The medical model has currently changed to the “biological-psycho-social” model [23]; people are no longer satisfied with simple life extension, but they have a higher hope for the quality of life. Therefore, the secondary efficacy in our study design, the indicators, mainly observe the improvement of patients’ symptoms and quality of life, including the secondary outcomes, such as angina classification (CCS), TCM syndromes, and Seattle Angina Scale score (SAQ) [24].

Our clinical design plan is based on the principles of international clinical trials. Strict quality control measures are designed using double-blind, randomized, placebo-controlled, multi-center research methods. A professional independent statistical group is responsible for statistical analysis and reporting, and it will analyze the combined data from all research centers. Strict trial design and supervision during the trial implementation process will ensure a scientific and objective evaluation of the efficacy and safety of SJP in the treatment of angina pectoris.

## Trial status

The final protocol version is 2.0 and is dated 18 February 2019. Recruitment began on 15 June 2019 and recruitment will be completed on 15 August 2020. This study has recruited 293 participants.

## Supplementary Information

**Additional file 1.** SPIRIT 2013 Checklist: Recommended items to address in a clinical trial protocol and related documents*.

## Data Availability

The datasets used and/or analyzed during the current study are available from the corresponding author on reasonable request.

## Abbreviations

SJP: Suxiao Jiuxin Pill
EKG: Electrocardiogram
CHD: Coronary heart disease
CVD: Cardiovascular disease
MACE: Major adverse cardiovascular events
APT: Antiplatelet therapy
ACEI: Angiotensin-converting enzyme inhibitors
ARB: Angiotensin II receptor blockers
GCP: Good Clinical Practice
TCM: Traditional Chinese medicine
MI: Myocardial infarction
CAD: Coronary artery disease
CCS: Canadian Cardiovascular Society
SBP: Systolic blood pressure
DBP: Diastolic blood pressure
FAS: Full analysis set
PPS: Per protocol set
SS: Safety set
ITT: Intention to treat
LOCF: Last observation carried forward
SD: Standard deviation
